# Risk factor analysis and a new prediction model of venous thromboembolism after pancreaticoduodenectomy

**DOI:** 10.1186/s12893-023-01916-9

**Published:** 2023-01-28

**Authors:** Zhi-Jie Yin, Ying-Jie Huang, Qi-Long Chen

**Affiliations:** Digestive and Vascular Center, Department of Pancreatic Surgery, The First Affiliated Hospital of Xinjiang Medical University, Urumqi, 830054 People’s Republic of China

**Keywords:** Pancreaticoduodenectomy, Venous thromboembolism, Prediction model, Nomogram

## Abstract

**Aim:**

The present study aimed to identify risk factors for venous thromboembolism (VTE) after pancreaticoduodenectomy (PD) and to develop and internally validate a predictive model for the risk of venous thrombosis.

**Methods:**

We retrospectively collected data from 352 patients who visited our hospital to undergo PD from January 2018 to March 2022. The number of patients recruited was divided in an 8:2 ratio by using the random split method, with 80% of the patients serving as the training set and 20% as the validation set. The least absolute shrinkage and selection operator (Lasso) regression model was used to optimize feature selection for the VTE risk model. Multivariate logistic regression analysis was used to construct a prediction model by incorporating the features selected in the Lasso model. C-index, receiver operating characteristic curve, calibration plot, and decision curve were used to assess the accuracy of the model, to calibrate the model, and to determine the clinical usefulness of the model. Finally, we evaluated the prediction model for internal validation.

**Results:**

The predictors included in the prediction nomogram were sex, age, gastrointestinal symptoms, hypertension, diabetes, operative method, intraoperative bleeding, blood transfusion, neutrophil count, prothrombin time (PT), activated partial thromboplastin time (APTT), aspartate aminotransferase (AST)/alanine aminotransferase (ALT) ratio (AST/ALT), and total bilirubin (TBIL). The model showed good discrimination with a C-index of 0.827, had good consistency based on the calibration curve, and had an area under the ROC curve value of 0.822 (P < 0.001, 95%confidence interval:0.761–0.882). A high C-index value of 0.894 was reached in internal validation. Decision curve analysis showed that the VTE nomogram was clinically useful when intervention was decided at the VTE possibility threshold of 10%.

**Conclusion:**

The novel model developed in this study is highly targeted and enables personalized assessment of VTE occurrence in patients who undergo PD. The predictors are easily accessible and facilitate the assessment of patients by clinical practitioners.

## Introduction

Pancreaticoduodenectomy (PD) is the primary treatment for benign and malignant tumors involving the head of the pancreas and ampulla of the duodenum and for severe pancreatic and duodenal injuries [1, 2]. It was first performed and reported by Whippe [3] in 1935. It is known as the ceiling of surgery because of the anatomical complexity of the surgical site, the wide range of the operative area, the difficulty of anastomosis, and the high anatomical and technical requirements for the surgeon. With the constant update of surgeons’ knowledge and advancements in surgical techniques, the safety of surgery has greatly improved, and the mortality rate associated with the surgery has gradually reduced, with a reported mortality rate of < 6% [4]; moreover, the scope of application has gradually widened. However, the postoperative complications of PD include postoperative pancreatic fistula, biliary fistula, hemorrhage, abdominal infection, delayed gastric emptying after surgery, and venous thrombosis [5], which lead to longer hospitalization days and higher hospitalization costs, thereby delaying patients’ recovery and increasing their financial burden.

Venous thromboembolism (VTE) is a common disease that mainly includes deep vein thromboembolism (DVT) and pulmonary embolism (PE), but it may also occur in visceral veins and cerebral veins [6]. Risk factors of the above diseases are common, including any factor can cause venous blood stasis, venous endothelial injury, and thrombophilia, are common and classified either hereditary or acquired. Among the hereditary risk factors, attention should be paid to factors that are relevant to embolic disorders.For instance,whether there are factor V leiden and prothrombin gene mutations,or deficiencies of protein C and antithrombin in the patients. Acquired risk factors are also more frequently observed. The identified risk factors include age, smoking, obesity, recent surgery, trauma, fracture, long-term bed rest, long-distance travel, acute infectious diseases, chronic obstructive pulmonary diseases, malignant tumors, cardiovascular and cerebrovascular diseases, hematological disorders, metabolic diseases, and rheumatic immune diseases [6–9], These risk factors may be present alone or in combination. Strong inducing factors for VTE include surgery and active cancer, but most events are unprovoked. Surgery is a common pathogenic factor of VTE, which may be due to the combination of vascular endothelial injury caused by surgery, hemodynamic changes, operation method, operation time, vascular reconstruction, blood transfusion during operation, and patients’ own factors. Cancer-associated thromboembolism (CAT) is associated with worse prognosis in cancer patients [10]. The incidence of VTE in cancer patients also depends on the type of cancer (pancreatic cancer, gastric cancer, and primary brain tumor have the highest risk) [11] as well as the patient’s own factors and cancer treatment-related factors.

Currently, most of the domestic and international VTE risk assessment scales are designed for surgical and orthopedic patients. However, when assessing the risk of VTE in surgical patients, not only patient-specific factors but also surgery-specific factors should be considered. There is, unfortunately, no similar scale specifically for patients undergoing PD. Surgeons mostly evaluate the risk of thromboembolism after PD on the basis of the Caprini scale [12, 13], the American College of Chest Physicians (ACCP) VTE prevention guidelines, and other available scoring models [14, 15] and empirically use limb pneumatic pumps, low-molecular-weight heparin, and other anticoagulant agents to prevent thrombosis. VTE occurs in patients undergoing PD because of many factors, and therefore, the preoperative and postoperative evaluation scales should be different. Postoperative patients are affected by many factors, such as the operation method, operation time, and other surgical factors. Moreover, the patient’s own factors could greatly increase the risk of VTE after PD. Hence, it is necessary to integrate the relevant VTE prevention and treatment guidelines and the characteristics of PD to establish a database of specific risk factors and acquired risk factors of PD patients and the interaction of various factors to improve the existing risk assessment model; this knowledge can then be used to establish a VTE score scale for PD patients, which can effectively differentiate high-, medium-, and low-risk VTE patients, provide accurate prediction, and enable individualized anticoagulant treatment. This approach can effectively reduce the incidence of VTE and bleeding after anticoagulation, thereby improving the quality of life and prognosis of PD patients to some extent.

The present study aimed to develop an effective and simple prediction tool on the basis of single factor and multifactor regression analyses, integrate multiple prediction indicators, and use nomograms to transform complex regression equations into visual graphs, which can directly reflect the predicted value of individual outcome events, that is, the probability of VTE. The purpose here is to provide a reliable judgment for the occurrence of VTE events after PD and, to a certain extent, to provide a reliable tool for clinicians to judge whether VTE prevention and intervention treatments are required; simultaneously, the prediction tool could facilitate to provide effective help for reducing adverse events such as VTE and bleeding after PD, thereby improving the quality of life and prognosis of PD patients to some extent.

## Patients and methods

### Patients and data collection

By using the case retrieval system, patients who underwent PD in the First Affiliated Hospital of Xinjiang Medical University from January 2018 to March 2022 were retrieved. The investigators included clinical staff of the Department of Pancreatic Surgery of the First Affiliated Hospital of Xinjiang Medical University and those with clinical experience after professional training. Patients treated for PD with a complete history and description of pathological findings and a clear imaging diagnosis of VTE in our ultrasound or radiology department were included in our study. We excluded (1) patients with a preoperative or previous history of VTE (n = 3), (2) patients with other recent surgical comorbidities (n = 1), (3) patients with severe liver and kidney diseases (n = 5), or (4) patients with hematological diseases and those with missing key clinical information (n = 0). Ultimately, 352 PD patients were included in this study. All data, including demographic, disease characteristics, and treatment characteristics, were collected.

### Statistical analysis

The number of patients recruited was divided randomly in the ratio of 8:2, of which 80% of the patients were used as the training set to establish the prediction model, and the remaining 20% of the patients were used as the validation set to verify the accuracy of the model. All data, including demographic, disease characteristics, and treatment characteristics, were expressed by count (%). The data were entered into an Excel spreadsheet. Statistical analysis was performed using SPSS version 26.0 software (IBM, Armonk, NY, USA) and the R studio software (version 4.2.1; https://www.rstudio.com).

The Least Absolute Shrinkage and Selection Operator (Lasso) [16, 17] method was suitable for shrinking high-dimensional data and filtering out the optimal predictive features in risk factors from PD patients with VTE. Characterised by variable selection and regularization in the same process as fitting a generalised linear model, Lasso regression selectively adjusts variables in models, especially those showing high levels of multicollinearity, to obtain optimal performance parameters and avoids overfitting (through regularization) at the same time.A multivariate logistic regression analysis was then used to construct a new predictive model by incorporating the features selected in the Lasso regression model. The features were regarded as odds ratio (OR) with 95% confidence interval (CI) and as P-value. Subsequently, a nomogram was plotted according to the results of logistic regression.

Calibration curves were drawn to assess the calibration of the VTE nomogram, which was then used to compare the consistency of the model prediction with the observed values. To estimate the discrimination capability of the VTE nomogram, the C-index and the area under the ROC curve were measured. The VTE nomogram was internally validated to calculate a relatively corrected C-index. Decision curve analysis was performed to determine the clinical usefulness of the VTE nomogram by quantifying the net benefit at different threshold probabilities. The net benefit was calculated by subtracting the proportion of true-positive patients from the proportion of false-positive patients among all patients and by weighing the relative harms of excluding the intervention against the negative consequences of an unnecessary intervention [18].

## Results

### Patients’ characteristics

**Fig. 1:**
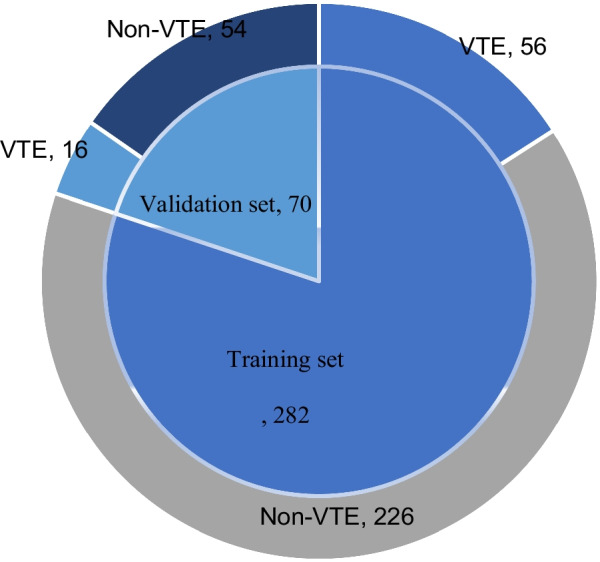
Composition of the two groups

Based on the defined inclusion criteria in this study, 352 patients with PD were included as subjects and categorised taking the similar rate of venous thromboembolism (VTE) in each group into consideration as follows: 56 of 282 patients (about 22.2%) in the training set developed VTE while approximately 22.8% in the validation set, that is, 16 of 70 patients, had been suffering this disease, as shown in Fig. 1.

We evaluated 43 candidate variables and compared each detection index between the training set and the validation set. The results confirmed that the training set and the validation set showed statistically significant differences in the blood group type, invasion outside the primary site, and PT% (P < 0.05), but no significant differences were observed in the remaining 40 variables, (P > 0.05). However, the blood group type, invasion outside the primary site, and PT% are laboratory indices and cannot represent the characteristics of the population; hence, they were not excluded in the follow-up study. Table 1 shows all data of the study patients, including the demographic, disease, and treatment features of the two groups.

**Table 1:** Comparison of general information of patients between the two groups

Demographic characteristics	Traning (n = 282)	Validation (n = 70)	P
Age (years)			
≦ 60	143 (50.71%)	39 (55.71%)	0.453
> 60	139 (49.29%)	31 (44.29%)	
Sex			
Female	50 (17.73%)	8 (11.43%)	0.203
Male	232 (82.26%)	62 (88.57%)	
Tumor site			
Head of pancreas	145 (51.41%)	31 (44.29%)	0.193
The ampulla of vater	68 (24.11%)	17 (24.29%)	
Duodenum	27 (9.58%)	13 (18.57%)	
Biliary tract	42 (14.90%)	9 (12.85%)	
Degree of tumor differentitation			
Benign	41 (14.54%)	12 (17.14%)	0.620
Highly differentitated	23 (8.16%)	4 (5.72%)	
Highly-moderately differentitated	12 (4.25%)	4 (5.72%)	
Moderately differentitated	103 (36.53%)	26 (37.14%)	
Moderately-poorly differentitated	77 (27.30%)	14 (20.00%)	
Poorly differentitated	26 (9.22%)	10 (14.28%)	
Blood type			
A	70 (24.82%)	26 (37.14%)	0.047
B	100 (35.46%)	17 (24.29%)	
O	68 (24.11%)	21 (30.00%)	
AB	44 (15.61%)	6 (8.57%)	
Pian			
No	169 (59.93%)	46 (65.71%)	0.374
Yes	113 (40.07%)	24 (34.29%)	
Jaundice			
No	139 (49.29%)	41 (58.57%)	0.164
Yes	143 (50.71%)	29 (41.43%)	
Lost weight			
No	111 (39.36%)	25 (35.71%)	0.589
Yes	171 (60.64%)	45 (64.29%)	
Gastrointestinal symptoms			
No	233 (82.62%)	52 (74.79%)	0.112
Yes	49 (17.38%)	18 (25.71%)	
Drinking history			
No	220 (78.01%)	52 (74.79%)	0.505
Yes	62 (21.89%)	18 (25.71%)	
Hypertension			
No	195 (69.15%)	49 (70.00%)	0.890
Yes	87 (30.85%)	21 (30.00%)	
Diabetes			
No	228 (80.85%)	59 (84.29%)	0.507
Yes	54 (19.15%)	11 (15.71%)	
Hepatitis B			
No	276 (97.87%)	68 (97.14%)	0.714
Yes	6 (2.13%)	2 (2.86%)	
Hepatitis C			
No	280 (99.29%)	69 (98.57%)	0.487
Yes	2 (0.71%)	1 (1.43%)	
Operative method			
PD	217 (76.95%)	56 (80.00%)	0.458
LPD	39 (13.83%)	6 (8.57%)	
LPD + PD	26 (9.22%)	8 (11.43%)	
Intraoperative bleeding (> 500 ml)			
No	157 (55.67%)	39 (55.71%)	0.995
Yes	125 (44.33%)	31 (44.29%)	
Operation time (> 600 min)			
No	184 (65.25%)	44 (62.86%)	0.708
Yes	98 (34.75%)	26 (37.14%)	
Blood transfusion			
No	169 (59.93%)	40 (57.14%)	0.671
Yes	113 (40.07%)	30 (42.86%)	
Minus jaundice measures			
No	224 (79.43%)	57 (81.43%)	0.710
Yes	58 (50.57%)	13 (18.57%)	
Vascular resection			
No	250 (88.65%)	64 (91.43%)	0.503
Yes	32 (11.35%)	6 (8.57%)	
History of abdominal surgery			
No	202 (71.63%)	54 (77.14%)	0.354
Yes	80 (28.37%)	16 (22.86%)	
Invasion outside the primary site			
No	172 (60.99%)	40 (57.14%)	0.012
Yes	110 (39.01%)	30 (42.86%)	
Tumor size (≧ 3 cm)			
No	134 (47.52%)	30 (42.86%)	0.484
Yes	148 (52.48%)	40 (57.14%)	
Blood vessel invasion			
No	163 (57.80%)	45 (64.29%)	0.323
Yes	119 (42.20%)	25 (35.71%)	
Nerve invasion			
No	103 (36.52%)	31 (44.29%)	0.231
Yes	179 (63.48%)	39 (55.71%)	
Lymphatic metastasis			
No	198 (70.21%)	51 (72.86%)	0.663
Yes	84 (29.79%)	19 (27.14%)	
R1 resection			
No	245 (86.88%)	50 (71.43%)	0.002
Yes	37 (13.12%)	20 (28.57%)	
Pancreatic duct dilatation			
No	122 (43.26%)	29 (41.43%)	0.781
Yes	160 (56.74%)	41 (58.57%)	
BMI (≧ 25 kg/m^2^)			
No	179 (63.48%)	42 (60.00%)	0.590
Yes	103 (36.52%)	28 (40.00%)	
HGB (≧ 120 g/L)			
No	101 (35.82%)	29 (41.43%)	0.384
Yes	181 (64.18%)	41 (58.57%)	
WBC (≦ 10 × 10^9^/L)			
No	28 (9.93%)	5 (7.14%)	0.474
Yes	254 (90.07%)	65 (92.86%)	
Neutrophil count (< 6.3 × 10^9^/L)			
No	55 (19.05%)	15 (21.43%)	0.718
Yes	227 (80.50%)	55 (78.57%)	
Lymphocyte count (< 3.2 × 10^9^/L)			
No	5 (1.77%)	1 (1.45%)	1
Yes	277 (98.23%)	69 (98.55%)	
PLT (10^9^/L)			
≦ 125	4 (1.42%)	*	0.092
125–350	234 (82.98%)	52 (74.79%)	
≧ 350	44 (15.60%)	18 (25.71%)	
CA19-9 (> 25 U/mL)			
No	119 (42.20%)	30 (42.86%)	0.920
Yes	163 (57.80%)	40 (57.14%)	
PT(s)			
≦ 9	7 (2.49%)	2 (2.86%)	0.855
9–15	263 (93.26%)	66 (94.28%)	
≧ 15	12 (4.25%)	2 (2.86%)	
APTT(s)			
≦ 23	1 (0.35%)	1 (1.43%)	0.546
23–38	258 (91.48%)	64 (91.43%)	
≧ 38	23 (8.16%)	5 (7.14%)	
PT%			
≦ 75	66 (23.40%)	7 (10.00%)	0.041
75–120	187 (66.31%)	53 (75.71%)	
≧ 120	29 (10.28%)	10 (14.28%)	
AST/ALT			
≦ 1	148 (52.48%)	42 (60.00%)	0.259
> 1	134 (47.52%)	28 (40.00%)	
Albumin (> 35 g/L)			
No	88 (31.21%)	28 (40.00%)	0.161
Yes	194 (68.79%)	42 (60.00%)	
TBIL (> 22 µmmol/L)			
No	91 (32.27%)	28 (40.00%)	0.221
Yes	191 (67.73%)	42 (60.00%)	
TG (≧1.69 mmol/L)			
No	126 (44.68%)	32 (45.71%)	0.876
Yes	156 (55.32%)	38 (54.29%)	
PCT (< 0.05 ng/mL)			
No	237 (84.04%)	62 (88.57%)	0.343
Yes	45 (15.96%)	8 (11.43%)	

*LPD* laparoscopic pancreatoduodenectomy, *PD *open pancreatoduodenectomy, *PT* prothrombin time, *BMI* body mass index, *HGB* hemoglobinopathies, *WBC *white blood cells, *PLT *platelet, *APTT *activated partial thromboplastin time, *AST/ALT *the aspartat aminotransferase (AST)/alanin aminotransferase (ALT) ratio, *TBIL *total bilirubin, *TG *triglyceride, *PCT *procalcitonin

### Feature selection

**Fig. 2:**
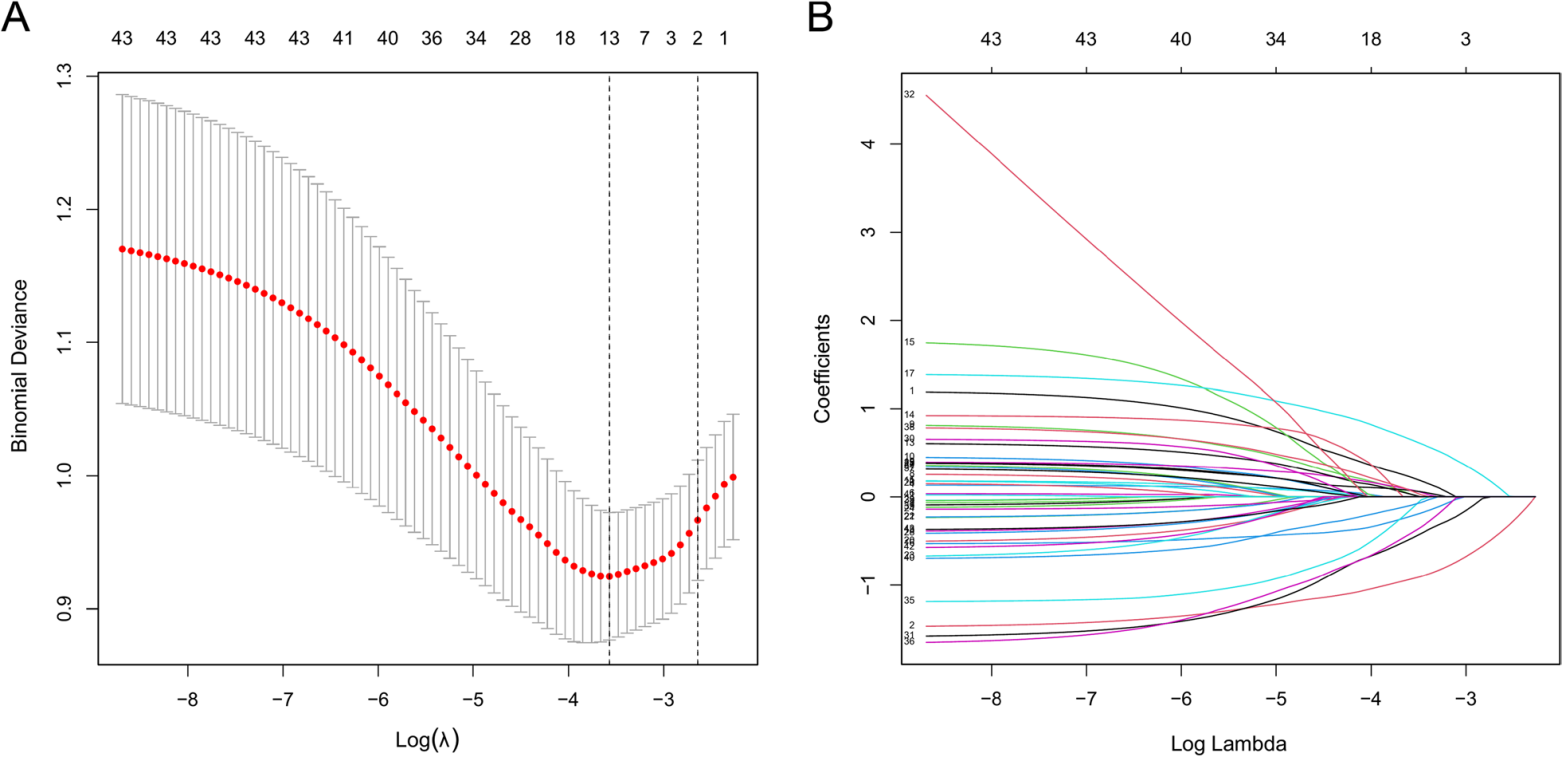
**A** Optimal parameter (lambda) selection in the Lasso model used five-fold cross-validation through the minimum criteria. Dotted vertical lines were drawn at the optimal values by using the minimum criteria and the 1 SE of the minimum criteria (the 1-SE criteria). **B** Lasso coefficient profiles of the 43 features. A coefficient profile plot was generated against the log(lambda) sequence. A vertical line was drawn at the value selected using five-fold cross-validation, where optimal lambda resulted in 13 features with nonzero coefficients. *Lasso* least absolute shrinkage and selection operator, *SE* standard error

In the training set, Lasso regression screened 13 potential predictors out of 43 candidate variables. These factors were characterized by nonzero coefficients in the Lasso regression model (Fig. 2A, B). These features included sex, age, gastrointestinal symptoms, hypertension, diabetes, operative method, intraoperative bleeding, blood transfusion, neutrophil count, PT, APTT, AST/ALT, and TBIL.

### Development of an individualized prediction model

**Fig. 3:**
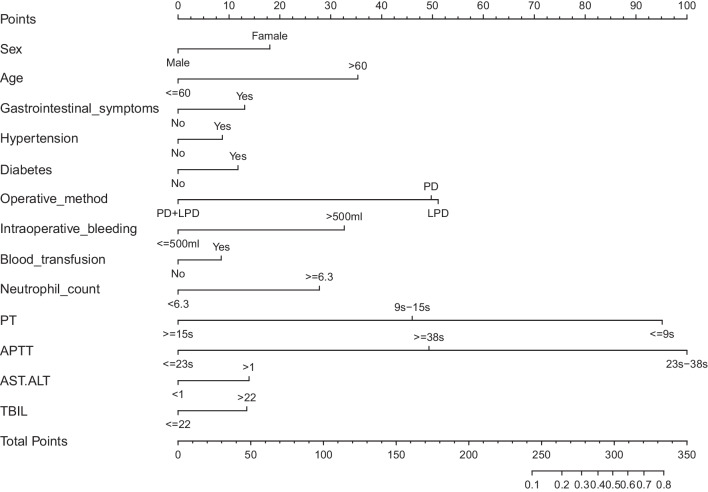
The Developed VTE nomogram

Table 2 shows the results of the logistic regression analysis for sex, age, gastrointestinal symptoms, hypertension, diabetes, operative method, intraoperative bleeding, blood transfusion, neutrophil count, PT, APTT, AST/ALT, and TBIL. A model that incorporated these independent predictors was developed and presented as the nomogram (Fig. 3).

**Table 2:** Prediction factors for VTE in patients undergoing pancreatoduodenectomy

Intercept and variable	Prediction model			95%CI	
	β	P-value	Odds ratio	Lower	Upper
Intercept	− 10.8864	0.9901	1.87E−05	NA	4.00E+60
Sex	0.7131	0.1018	2.04	0.859	4.798
Age	− 1.3973	0.0003	2.472	0.111	0.52
Gastrointestinal symptoms	0.5174	0.2401	1.677	0.695	3.953
Hypertension	0.3451	0.3705	1.412	0.658	3.003
Diabetes	0.4653	0.2724	1.593	0.684	3.639
Operative method					
LPD	0.0553	0.9186	1.056	0.345	2.959
PD + LPD	− 1.9675	0.0669	0.139	0.007	0.771
Intraoperative bleeding	1.2920	0.0005	3.64	1.771	7.787
Blood transfusion	0.3361	0.3658	1.399	0.673	2.909
Neutrophil count	− 1.0978	0.0069	0.334	0.149	0.742
PT(s)					
9–15	− 1.9420	0.1871	0.143	0.008	4.207
≧ 15	− 3.7631	0.0514	0.023	0.0004	1.222
APTT(s)					
23–38	11.7717	0.9893	1.30E+05	1.83E−61	NA
≧ 38	9.7665	0.9911	1.74E+04	4.88E−53	NA
AST/ALT	0.5509	0.1224	1.734	0.868	3.537
TBIL	− 0.5344	0.1618	0.589	0.275	1.243

β is the regression coefficient

*CI *confidence interval, *LPD* laparoscopic pancreatoduodenectomy, *PD* open pancreatoduodenectomy, *PT* prothrombin time, *APTT* activated partial thromboplastin time, *AST/ALT* The aspartat aminotransferase (AST)/alanin aminotransferase (ALT) ratio, *TBIL* total bilirubin

### Performance of the VTE risk nomogram in the study cohort

**Fig. 4:**
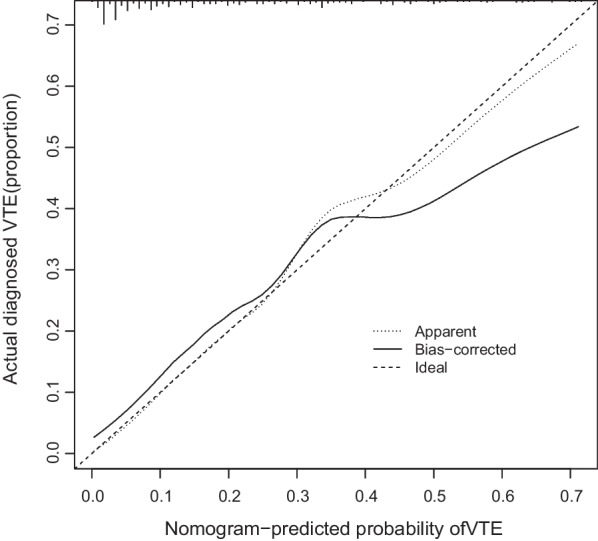
Calibration curves of the VTE prediction nomogram. The x-axis represents the predicted medication nonadherence risk. The y-axis represents the actual diagnosed nonadherence. The diagonal dotted line represents a perfect prediction by an ideal model. The solid line represents the performance of the nomogram, and a closer fit to the diagonal dotted line represents a better prediction

**Fig. 5:**
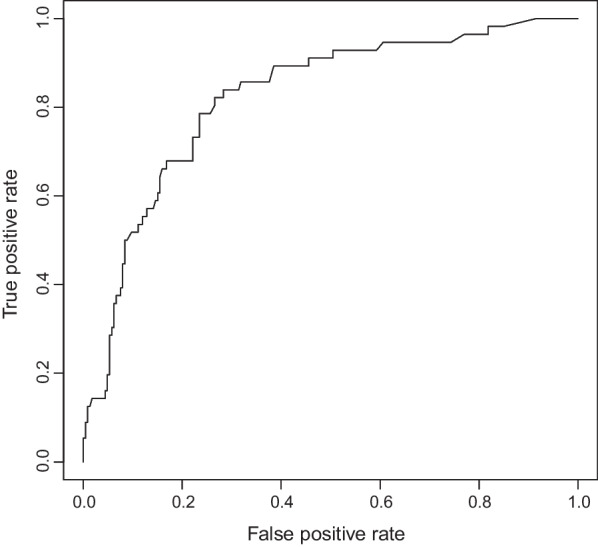
The area under the receiver operating characteristic curve (AUC) for the prediction nomogram. The ROC curve was measured to discriminate the performance of the VTE nomogram. The area under the receiver operating characteristic (ROC) curve (AUC) for the prediction nomogram was 0.822 (P < 0.001, 95%confidence interval:0.761–0.882), with a sensitivity of 0.839 and a specificity of 0.717

The calibration curve of the VTE risk nomogram for the prediction of VTE risk in patients undergoing PD showed a good agreement in the training set (Fig. 4). The area under the receiver operating characteristic curve (AUC) for the prediction nomogram was 0.8218 (P < 0.001, 95%confidence interval:0.761–0.882), with a sensitivity of 0.839 and a specificity of 0.717(Fig. 5), which showed that the prediction model had good accuracy. The C-index for the prediction nomogram was 0.827 for the training set, and it was confirmed to be 0.894 through internal validation; this finding suggested that the model had good discrimination ability. Thus, the performance of the VTE risk nomogram indicated that it had a good prediction capability.

### Clinical application

**Fig. 6:**
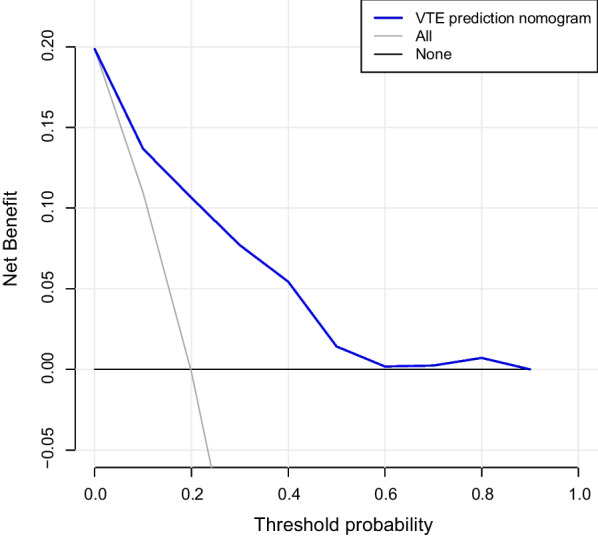
Decision curve analysis for the VTE prediction nomogram. The y-axis represents the net benefit. The blue line indicates the VTE risk Nomogram: the thin line represents all PD patients who developed VTE, while the thick line represents all PD patients who did not develop VTE

The decision curve analysis for the medication nonadherence nomogram is presented in Fig. 6. The decision curve showed that if the threshold probability of a PD patient was > 10%, then the use of this VTE nomogram to predict VTE risk adds more benefit than the scheme. Within this range, the net benefit was comparable with several overlaps on the basis of the VTE risk nomogram.

The decision curve shows that the predictive model has a high clinical application if the threshold probability for PD patients is > 10%, respectively.

## Discussion

In our present study, we combined the relevant domestic and international guidelines for the prevention and treatment of VTE after PD. According to the characteristics of PD, 43 candidate variables were chosen. Of these, 13 variables with nonzero coefficient characteristics were screened out by Lasso regression. These 13 variables were integrated, and nomograms were drawn accordingly. The complex regression equations were transformed into visual graphs to visually reflect the predicted value of individual outcome events, that is, the probability of VTE. The model evaluation showed the following results: C-index was 0.827, the calibration curve showed good consistency, the AUC of the ROC curve was 0.822 (P < 0.001, 95%confidence interval:0.761–0.882), with a sensitivity of 0.839 and a specificity of 0.717, and the internal validation C-index was 0.894. These indicators showed that the model was relatively accurate. The prediction model could provide a reliable judgment for the occurrence of VTE events after PD. The model also achieved individualized predictions, and thus, clinicians can use this tool to assess patients for their participation in clinical trials. Moreover, this study was the first to apply a nomogram to predict VTE after PD.

The risk factor analysis revealed that female gender, age > 60 years, presence of preoperative gastrointestinal symptoms, complications of hypertension and diabetes, use of laparoscopic pancreaticoduodenectomy (LPD) as the operation method, intraoperative hemorrhage > 500 mL and blood transfusion; preoperative neutrophil count ≥ 6.3 × 10^9^/L; PT ≤ 9 s, APTT of 23–38 s, AST/ALT > 1, and TBIL > 22 µmol/L may be the risk factors for VTE in patients undergoing PD.

In this study, we found that the female gender might be a risk factor for VTE after PD. A significant difference is observed in the patient composition ratio between male and female patients, and this finding may be attributed to the high incidence of pancreatic cancer and other diseases that require treatment with PD in males [19, 20]. Therefore, the prevalence rate of VTE in the two groups was compared. As shown in Table 3, the prevalence rate of VTE in female patients in the training and validation sets was 30% and 37.5%, respectively, which was much higher than those for male patients.

**Table 3:** Comparison of the prevalence of VTE in sex between two groups

	Training set			Validation set		
	VTE	Non-VTE	Prevalence rate (%)	VTE	Non-VTE	Prevalence rate (%)
Female	15	35	30.0	3	5	37.5
Male	41	191	17.7	6	56	9.7
Total	56	226	19.8	9	61	12.8

Previous studies [8] have shown that the incidence rate of VTE increases markedly with age for both male and female patients and for both DVT and PE. The incidence rates are somewhat higher in females during childbearing years, while the incidence rates are generally higher in males after the age of 45 years; thus, the incidence rate may vary by gender according to age and exposure to gender-specific triggers [21]. Interestingly, a large cohort study [22] in Sweden found that males have a higher incidence of first-ever VTE than females. Risk factors for VTE may differ between male and female patients. The Swedish cohort study also found that hypertension seems to have a protective effect on the future incidence of VTE in males, but not in females. Our present study showed that hypertension might be a risk factor for postoperative VTE, and in agreement with the Swedish cohort study, male patients with hypertension had a lower risk of future incidence of VTE, while female patients with hypertension had a significantly higher risk for VTE than male patients. This finding indicates that hypertension may play different roles according to gender in patients with VTE, and future research should focus on understanding this difference in detail.

We hypothesized that the choice of surgical method, the amount of intraoperative bleeding, and the volume of blood transfusion are risk factors for the development of VTE after PD. We found that the choice of LPD as the surgical method may be a risk factor for postoperative VTE in PD patients, while conversion to open laparoscopic surgery (LPD + PD) was not a risk factor for postoperative VTE in PD patients; this finding may be related to the longer duration of LPD and compromised blood return due to increased abdominal pressure from the establishment of the pneumoperitoneum. In a retrospective study, Brent et al. [23] found that as compared to open distal pancreatectomy, minimally invasive distal pancreatectomy was associated with a higher incidence of postoperative VTE in patients. However, no similar studies have reported the relationship between the choice of surgical approach for PD and the development of VTE after PD. Furthermore, our study reached the same conclusion as previous studies [24, 25] that perioperative RBC transfusion may be significantly related to the development of new or progressive postoperative VTE, independent of several hypothetical confounding factors. These findings, if confirmed, should reinforce the significance of strict management of perioperative transfusion practices. Heavy intraoperative bleeding is correlated with blood transfusion; this clearly indicates that intraoperative bleeding is a risk factor for VTE after PD. This implies that surgeons should closely monitor such patients. Moreover, because abdominal bleeding is also a common postoperative complication of PD, it is clear that the development of VTE is a cumulative effect of various factors. In summary, surgical factors play a crucial role in the development of VTE in PD patients postoperatively.

There is an increasing consensus that thromboembolism is closely linked to inflammation, and it is possible that inflammation is involved in the initiation of thrombosis. We also found that preoperative neutrophil counts may be relevant to the formation of VTE after PD. Mauracher et al. [26] showed that neutrophils may enhance thromboembolism by releasing neutrophil extracellular traps (NETs) to enhance thrombosis. In addition, activated neutrophils release proteases that degrade anticoagulant tissue factor (TF) pathway inhibitors, thereby increasing TF activity and activation of the coagulation pathway. Plasma guanosine levels of plasma citrullinated histone H3, a biomarker of NET formation, are associated with VTE in patients with pancreatic cancer (PC). Elevated neutrophil count is a non-specific biomarker of inflammation [27]. A multicentre retrospective study [28] showed that postoperative intraperitoneal infection increased the risk of Splanchnic vein thrombosis (SVT). The study also drew our attention to the fact that local inflammation played an important role in the development of SVT, which was associated with mortality at 90 days postoperatively. In summary, inflammation may be a precursor of thrombosis; hence, anti-inflammatory treatment may be a protective factor for VTE and could provide new strategies for thromboembolism prevention. This hypothesis needs to be confirmed in prospective studies.

Obstructive jaundice [29] is a common clinical symptom of a lesion occupying the head of the pancreas, lower bile duct, and duodenal papilla. In the present study, we included TBIL, PT, and APTT, which are indicators of liver function to a certain extent, to investigate whether these factors could be used as predictors of VTE after PD. We found that elevated TBIL level was associated with postoperative VTE development in PD patients. Interestingly, in other studies [30], bilirubin was found to have anti-inflammatory effects along with antioxidant effects. A retrospective case–control study by Cervellin et al. [31] established a significant relationship between low TBIL levels and PE. Mustafa et al. [32] showed that the presence of a genetic variant of the heme oxygenase 1 gene increased the risk of VTE recurrence. Bilirubin inhibits collagen-induced platelet activation, vascular smooth muscle cell migration and proliferation, platelet aggregation, and neoplastic endothelial formation. Does this indicate that we obtained a wrong result? We, however, are not convinced that our result might be inappropriate. For example, although bilirubin has anti-inflammatory properties and inhibits platelet aggregation, it is undeniable that most patients undergoing PD have obstructive jaundice, and some of these patients often have varying levels of elevation of the tumor marker CA19-9. The tumor marker CA19-9 has been shown to predict the incidence of VTE in PC patients [33]. This may be related to the fact that elevated CA19-9 level may activate platelets and neutrophils, among other mechanisms, to activate the coagulation pathways and increase blood hypercoagulability. This indicates that VTE events occur following the interaction of multiple factors. Further experiments are required to verify whether bilirubin plays a protective or promotional role in the development of VTE in PD patients.

Our study is the first to use AST/ALT to assess the occurrence of VTE. AST and ALT are well-known circulating biomarkers for reflecting liver injury. The AST/ALT ratio is also known as the De Ritis ratio [34]. It was first proposed for studying the etiology of hepatitis and is often used to differentiate between the different causes of liver diseases, such as the fatty liver. Currently, the AST/ALT ratio is used as a validated biomarker for nonliver diseases such as cardiovascular diseases, cancer, type 2 diabetes, and other diseases [35, 36]. The AST/ALT ratio reflects impaired liver function; however, its elevation does not necessarily accurately reflect the severity of liver diseases. Hepatic impairment leads to a decrease in the levels of coagulation factors II, V, VII, IX, and X. This reduces coagulation and predisposes to bleeding tendencies. This is, however, contradictory to the results of our present study. The increase in the De Ritis ratio, on the one hand, increases the viscosity of the blood, and on the other hand, indicates the possibility of inflammation; thus, these parameters are associated with an increased risk of VTE. The AST/ALT ratio is an economical and readily available laboratory indicator that can be used in clinical monitoring of postoperative VTE in PD patients and also demonstrates the feasibility of our predictive model.

The shortened activated partial thromboplastin time (APTT) and prothrombin time (PT) are increasingly and frequently referred to as potential risk factors [37] for VTE. But our nomogram showed that this generally acknowledged point of view may be biased to a certain extent. In our study, the PT shortening was shown to be associated with the development of VTE whereas APTT was found to be the opposite. It seemed that APTT shortening is incorrelate with the development of VTE. The multivariate analysis demonstrated a comparatively wide confidence interval for APTT, which we considered as a consequence of the insufficient sample size, as shown in Table 4. However, we did not integrate the groups because APTT and VTE directly reflect the abnormalities in the coagulation system. The APTT has been used clinically to monitor the efficacy of anticoagulation [38], but whether prolongation of APTT is an absolute contraindication to anticoagulation deserves contemplation. As stated by Bachler et al. [39], the prolongation of APTT may lead to the deficient anticoagulation amount prescribed or even the absence of anticoagulation therapy, so the clinical significance of the APTT must be re-evaluated.

**Table 4:** Comparison of the prevalence of VTE in PT and APTT between two groups

	Training set		VTE (%)	Validation set		VTE (%)
	VTE	Non-VTE		VTE	Non-VTE	
PT(s)						
≦ 9	1	2	33.3	2	0	100.0
9–15	54	213	20.2	14	52	21.2
≧ 15	1	11	7.7	0	2	0.0
APTT(s)						
≦23	0	1	0.0	1	0	100.0
23–38	55	203	21.3	14	50	21.9
≧ 38	1	22	4.5	1	4	20.0

The essential goal of surgical extirpation of cancer is to achieve R0 resection, as margin-positive specimens are associated with poor long-term survival [40]. In addition, positive margins are strongly associated with disease recurrence and metastasis [41]. Therefore, we included the surgical resection margin as one of the factors to be investigated in the study and compared the prevalence rate of VTE in the two groups.. As shown in Table 1, the composition ratios of the two groups are statistically different (P < 0.05). When Lasso was applied to filter variables, surgical margins failed to remain their correlation with VTE. However, the retrospective nature of this study could not allow us to assume incoherence with the occurrence of VTE. Previous studies [42, 43] were keen to explore the relationships between positive surgical margins and overall survival, for instance, the study performed by Eurola et al. [42], which unfortunately discovered no associations in their univariate analysis. Although the results were not satisfactory, this might provide a new path for future large-population prospective studies.

Thus, VTE after PD occurs due to a combination of various factors. Hence, a relatively accurate assessment will help physicians to determine the probability of VTE in patients and to intervene in a timely manner to avoid adverse outcomes. The development of models for surgery-related VTE is a reflection of individualization and precision, which is a future trend. There are, however, several risk factors for VTE; therefore, the determination of the underlying cause is the key to prevent VTE events, and this aspect will be the topic of future research.

### Limitations

Our present study has several limitations. First, our study is retrospective in nature and cannot eliminate bias in the data, the sample size was small and did not achieve a minimum of 10 patients for each risk factor. Second, our data are obtained from a single institution. Thus, the obtained data are not representative of all Chinese PD patients. Third, data collection was incomplete, and the risk factor analysis did not include all potential factors affecting VTE. Finally, although we assessed several variables, the effects of undetermined confounders cannot be ignored. To overcome these limitations, external validation and prospective multicenter studies with a large number of patients are required.

## Conclusion

In summary, the predictors required for the model developed in the present study are clinically accessible; moreover, the previous complex regression equations have been transformed into intuitive graphs, thus making the predictive model more readable and convenient for clinicians to assess patients. The application of the model needs to be validated in future prospective studies.

## Data Availability

The datasets generated and analysed during the current study are not publicly available due to patient privacybut are available from the corresponding author on reasonable request.

## References

[1] Ban D (2022). International Expert Consensus on Precision Anatomy for minimally invasive distal pancreatectomy: PAM-HBP Surgery Project. J Hepatobil Pancreat Sci.

[2] Mihaljevic AL (2021). Not all Whipple procedures are equal: proposal for a classification of pancreatoduodenectomies. Surgery.

[3] Whipple AO (1945). Pancreaticoduodenectomy for islet carcinoma : a five-year follow-up. Ann Surg.

[4] Are C, Dhir M, Ravipati L (2011). History of pancreaticoduodenectomy: early misconceptions, initial milestones and the pioneers. HPB (Oxford).

[5] Kokkinakis S (2022). Complications of modern pancreaticoduodenectomy: a systematic review and meta-analysis. Hepatobil Pancreat Dis Int HBPD INT.

[6] Khan F, Tritschler T, Kahn SR, Rodger MA (2021). Venous thromboembolism. The Lancet.

[7] Cohen D (2021). Current issues in venous thromboembolism. Postgrad Med.

[8] Heit JA, Spencer FA, White RH (2016). The epidemiology of venous thromboembolism. J Thromb Thrombolysis.

[9] Paul JD, Cifu AS (2019). Prevention and management of venous thromboembolism. JAMA.

[10] Khorana AA (2022). Cancer-associated venous thromboembolism. Nat Rev Dis Primers.

[11] Hisada Y, Mackman N (2017). Cancer-associated pathways and biomarkers of venous thrombosis. Blood.

[12] Lu X (2021). Application of the Caprini risk assessment model for deep vein thrombosis among patients undergoing laparoscopic surgery for colorectal cancer. Medicine.

[13] Sterbling HM (2018). Caprini risk model decreases venous thromboembolism rates in thoracic surgery cancer patients. Ann Thorac Surg.

[14] Stevens, H., Peter, K., Tran, H. & McFadyen, J. Predicting the risk of recurrent venous thromboembolism: current challenges and future opportunities. *J Clin Med* 2020;9. 10.3390/jcm9051582. 10.3390/jcm9051582 PMC7290951 32456008

[15] Godinho J (2020). ONKOTEV score as a predictive tool for thromboembolic events in pancreatic cancer-a retrospective analysis. Oncologist.

[16] Venkatesh KK (2020). Machine learning and statistical models to predict postpartum hemorrhage. Obstet Gynecol.

[17] van Egmond MB (2021). Privacy-preserving dataset combination and Lasso regression for healthcare predictions. BMC Med Inform Decis Mak.

[18] Wang H (2018). Predicting medication nonadherence risk in a Chinese inflammatory rheumatic disease population: development and assessment of a new predictive nomogram. Patient Prefer Adherence.

[19] Lippi G, Mattiuzzi C (2020). The global burden of pancreatic cancer. Arch Med Sci AMS.

[20] Siegel RL, Miller KD, Fuchs HE, Jemal A (2022). Cancer statistics, 2022. CA Cancer J Clin.

[21] Faioni EM, Zighetti ML, Vozzo NP (2018). Sex, gender and venous thromboembolism: do we care enough?. Blood Coagul Fibrinol Int J Haemos Thromb.

[22] Lind MM, Johansson M, Själander A, Johansson L (2022). Incidence and risk factors of venous thromboembolism in men and women. Thromb Res.

[23] Willobee BA (2020). Minimally invasive surgery is associated with an increased risk of postoperative venous thromboembolism after distal pancreatectomy. Ann Surg Oncol.

[24] Goel R (2018). Association of perioperative red blood cell transfusions with venous thromboembolism in a north American Registry. JAMA Surg.

[25] Sheth BK (2022). Red blood cell transfusions and risk of postoperative venous thromboembolism. J Am Acad Orthop Surg.

[26] Mauracher LM (2018). Citrullinated histone H3, a biomarker of neutrophil extracellular trap formation, predicts the risk of venous thromboembolism in cancer patients. J Thromb Haemost.

[27] Hu J, Cai Z, Zhou Y (2022). The association of neutrophil-lymphocyte ratio with venous thromboembolism: a systematic review and meta-analysis. Clin Appl Thromb Hemost.

[28] Duceppe E (2021). Incidence and predictors of splanchnic vein thrombosis and mortality following hepatobiliary and pancreatic surgery. J Thromb Haemost.

[29] Satoh D, Matsukawa H, Shiozaki S (2022). The optimal type and management of biliary drainage in patients with obstructive jaundice who undergo pancreaticoduodenectomy. In Vivo (Athens, Greece).

[30] Yao Q (2019). Bilirubin improves the quality and function of hypothermic preserved islets by its antioxidative and anti-inflammatory effect. Transplantation.

[31] Cervellin G, Buonocore R, Sanchis-Gomar F, Lippi G (2016). Low serum bilirubin values are associated with pulmonary embolism in a case–control study. Clin Chem Lab Med (CCLM).

[32] Mustafa S (2008). Genetic variation in heme oxygenase 1 (HMOX1) and the risk of recurrent venous thromboembolism. J Vasc Surg.

[33] Peippo MH, Kurki S, Seppänen H, Lassila R, Carpén O (2020). CA 19-9 doubling time in pancreatic cancer as a predictor of venous thromboembolism: a hospital database study. Acta Oncolog (Stockholm, Sweden).

[34] Liu H (2021). AST/ALT ratio and peripheral artery disease in a Chinese hypertensive population: a cross-sectional study. Angiology.

[35] Chen W (2022). Elevated AST/ALT ratio is associated with all-cause mortality and cancer incident. J Clin Lab Anal.

[36] Zhou J, He Z, Ma S, Liu R (2020). AST/ALT ratio as a significant predictor of the incidence risk of prostate cancer. Cancer Med.

[37] Ono R, Fukushima K, Yamazaki T, Takahashi H, Hori Y (2020). The distribution of anti-factor Xa activity value, PT and APTT at peak and trough times in patients with direct anti-factor Xa inhibitors. Eur Heart J.

[38] Toulon P, Smahi M, De Pooter N (2021). APTT therapeutic range for monitoring unfractionated heparin therapy. Significant impact of the anti-Xa reagent used for correlation. J Thromb Haemost.

[39] Bachler M (2019). Influence of factor XII deficiency on activated partial thromboplastin time (aPTT) in critically ill patients. J Thromb Thrombolysis.

[40] Tempero MA (2021). Pancreatic Adenocarcinoma, Version 2.2021, NCCN Clinical Practice Guidelines in Oncology. J Nat Comprehen Cancer Netw JNCCN.

[41] Li B (2022). Risk factors of positive resection margin differ in pancreaticoduodenectomy and distal pancreatosplenectomy for pancreatic ductal adenocarcinoma undergoing upfront surgery. Asian J Surg.

[42] Eurola A (2022). Preoperative oncologic therapy and the prolonged risk of venous thromboembolism in resectable pancreatic cancer. Cancer Med.

[43] Schmocker RK (2021). Impact of margin status on survival in patients with pancreatic ductal adenocarcinoma receiving neoadjuvant chemotherapy. J Am Coll Surg.

